# The Bittersweet Sounds of the Modern Food Chain

**DOI:** 10.1371/journal.pbio.0040047

**Published:** 2006-02-14

**Authors:** Gavin Yamey

## Abstract

Matthew Hebert's new album *Plat du Jour* will make you question what you put on your plate.

The legendary chef Alice Waters, owner of the 34-year-old restaurant Chez Panisse in Berkeley, California, and the “godmother” of the organic-food movement, once famously served her customers a single peach on a plate for dessert. There was no accompanying cream or syrup. It wasn't even peeled or sliced. It was just a locally produced, seasonal fruit grown without pesticides that was—by all accounts—utterly delicious.[Fig pbio-0040047-g001]

**Figure pbio-0040047-g001:**
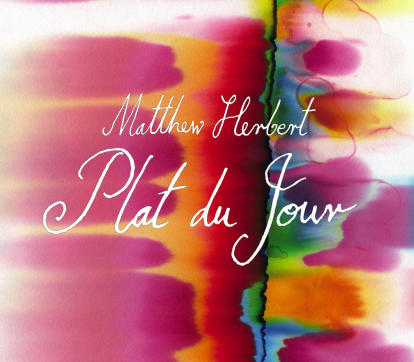


The British electronic musician and composer Matthew Herbert would surely have approved of Waters' gesture. Herbert's new album, *Plat du Jour* ( http://www.platdujour.co.uk), is both a musical critique of the social, environmental, and public-health impact of fast food (including the foods that are mass produced for quick consumption at home) and a rallying call for a simpler, slower, and healthier way of eating. After two years of detailed research and six months of sampling food-related sounds—such as the chirping of 30,000 broiler chickens and the puncturing of a Nescafé instant coffee jar—Herbert has created twelve tunes that he hopes will make you question what you put on your plate.

These tunes are best appreciated while simultaneously reading their “ingredients,” listed in the album's liner notes and on the Web site. The opening track, for example, called “the truncated life of a modern industrialized chicken” (a free sample of the tune is at http://www.platdujour.co.uk/downloads.php), contains recordings of “24,000 one minute old chicks in one room of a commercial hatchery, 40 free-range chickens in a coop, [and] one of those chickens being killed for a local farmer's market and its feathers washed and plucked.” It's not exactly easy listening.

In his interviews with the press, Herbert has explained that every decision he made when producing *Plat du Jour*—from the choice of field recordings to the length of each song—was aimed at provoking listeners to rethink their relationship with food. The striking multicolored cover art, for example, which resembles tie-dye, was created from synthetic food colorings dripped onto chromatography paper. The artwork is accompanied by a list of each coloring's chemical ingredients and the side effects of ingestion. The artist, Stanley Donwood, says he wanted to make artwork that mimicked the experience of walking into a brightly lit supermarket: “I wanted to make something beautiful, seductive, and entrancing, but which became slightly repellant when examined closely.”

It's not exactly easy listening.

This description fits much of the music itself. On first listening, the tunes are also entrancing, in part because their melodies and percussion were created by some of Britain's finest jazz musicians playing, among other things, Coke cans, chopsticks, and pickle jars. But the devil is in the details: once you discover Herbert's thoughtful reasoning behind each tune, you are left feeling somewhat queasy.

A track called “these branded waters,” for example, sounds rather upbeat, until you find out that it is a critique of the inequitable global distribution of safe drinking water and sanitary services. The track is exactly five minutes and 30 seconds long. Why? Because, the liner notes say, “Sanitation coverage is 53% in Bangladesh.” The track speed is 182 beats per minutes, because “it takes 182,000 litres of water to make one ton of steel.” The “instruments” used to create the track included a variety of brands of bottled water, the world's fastest-selling soft drink (the bottled-water industry is currently worth about US $46 billion worldwide). There is a troubling irony in the fact that while dirty drinking water is a major childhood killer in poor countries, many citizens in rich countries—where tap water is safe to drink—choose to pay up to 10,000 times more for branded water. The liner notes remind us that bottled waters are often owned or distributed by multinational corporations, such as Nestlé and Coca-Cola, which have regularly been criticized for disregarding environmental and public-health concerns. Bottled water also contributes 1.5 million tons to landfill sites each year.

Throughout *Plat du Jour*, Herbert uses this same trick of wrong-footing the listener in order to cajole, amuse, unsettle, and ultimately educate. At first, “an apple a day” seems to be a celebratory tune championing organic farm techniques. It is, after all, composed of the joyful sounds of more than 3,000 people crunching on locally grown, organic apples. But as we listen to the orchestrated munching, we read that although the United Kingdom grows more than 2,000 apple varieties, the nation's supermarkets are packed instead with apples imported—at huge environmental cost—from thousands of miles away.

Herbert again plays tricks with us on a rhythmic and jaunty track called “sugar,” created by recording chefs from Britain's world-renowned restaurant The Fat Duck, while they tunefully burn, melt, whisk, and boil sugar. This was no ordinary sugar: it was a European Union–subsidized sugar that had been “dumped in the world markets, artificially depressing global prices and having a particularly dire impact on former slave colonies of the Caribbean, established in the first place to grow, er … sugar.” The initial sweetness of the tune gives way to a bitter aftertaste.

At times, experiencing *Plat du Jour* feels a little like being at a lecture, rather than a concert hall. But the dazzling array of topics, from the celebrity marketing of unhealthy foods to the worryingly high levels of pesticide residues in mass-produced brown bread, together with Herbert's playfulness, prevent the album from being a dry polemic. One of the effects of “digesting” these songs is that they plant a seed of curiosity in the listener to find out more. Herbert helps us along in this process by providing links to relevant Web sites, a reading list of books on food production, and some longer online essays. These essays include an article by Norman Church on the effects of oil consumption on our food chain, as well as extracts from Antony Wild's book *Black Gold*, which discusses the coffee industry's dubious historical links with slavery and colonialism. [Fig pbio-0040047-g002]

**Figure pbio-0040047-g002:**
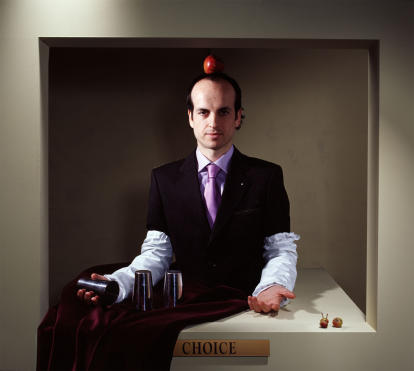
Our food choices, Herbert argues, can have a social, environmental, and public-health impact (Photo: Helen Woods)

Not surprisingly, after making *Plat du Jour* Herbert vowed to change his own ways. He has promised, for example, to reduce his air travel from once a week to once a year in order to reduce his personal contribution toward carbon emissions. He is also putting his money where his mouth is by donating a portion of the profits from the album to Navdanya ( http://www.navdanya.org), a biodiversity and seed-conservation movement in India. Herbert champions the group's work in a track called “the nine seeds of navdanya.” In the liner notes he explains that the Texan agribusiness company RiceTec was granted patent number 5663484 to patent basmati rice, a patent that Navdanya believes is a form of “biopiracy” (see http://www.navdanya.org/articles/basmati_biopiracy.htm). “Consequently,” Herbert writes, “I used the following numbers of seeds sent to me by navdanya to generate the sounds for this track: five basmati seeds, six mustard seeds, six millet seeds, three horse gram seeds, four barley seeds, eight wild cow pea seeds, [and] four green gram seeds.”

*Plat du Jour* follows in a recent line of popular exposés of the fast-food industry, most notably Morgan Spurlock's multi-award-winning movie *Super Size Me* ( http://www.supersizeme.com) and Eric Schlosser's New York Times bestseller *Fast Food Nation*. “Hundreds of millions of people buy fast food every day,” Schlosser writes, “without giving it much thought. They rarely consider where this food came from, how it was made, what it is doing to the community around them.” The enormous success of fast food is based on the promise of low cost to the consumer but, Schlosser says, “the real price never appears on the menu.” On *Plat du Jour*, a hugely ambitious musical experiment, it is the hidden costs of fast-food production that Herbert has succeeded in highlighting. I suspect that even if just one listener changes his or her eating habits, perhaps choosing for dessert a locally grown organic peach instead of a McDonald's Baked Apple Pie, Herbert will feel that his culinary efforts have been rewarded.

